# Expression of Cathepsins B, D, and G in Isocitrate Dehydrogenase-Wildtype Glioblastoma

**DOI:** 10.3389/fsurg.2017.00028

**Published:** 2017-05-29

**Authors:** Sabrina P. Koh, Agadha C. Wickremesekera, Helen D. Brasch, Reginald Marsh, Swee T. Tan, Tinte Itinteang

**Affiliations:** ^1^Gillies McIndoe Research Institute, Newtown, Wellington, New Zealand; ^2^Department of Neurosurgery, Wellington Regional Hospital, Wellington, New Zealand; ^3^Wellington Regional Plastic, Maxillofacial and Burns Unit, Hutt Hospital, Wellington, New Zealand

**Keywords:** glioblastoma, multiforme, cathepsin, cancer stem cells, renin-angiotensin system, bypass loop, isocitrate dehydrogenase, wildtype

## Abstract

**Aim:**

To investigate the expression of cathepsins B, D, and G, in relation to the cancer stem cell (CSC) subpopulations, we have previously characterized within isocitrate dehydogenase (IDH)-wildtype glioblastoma (IDHWGB).

**Methods:**

3,3-Diaminobezidine (DAB) immunohistochemical (IHC) staining for cathepsins B, D, and G, was performed on 4μm-thick formalin-fixed paraffin-embedded IDHWGB samples obtained from six patients. Two representative DHWGB samples from the original cohort of patients were selected for immunofluorescent (IF) IHC staining, to identify the localization of the cathepsins in relation to the CSC subpopulations. NanoString gene expression analysis and colorimetric *in situ* hybridization (CISH) were conducted to investigate the transcriptional activation of genes encoding for cathepsins B, D, and G. Data obtained from cell counting of DAB IHC-stained slides and from NanoString analysis were subjected to statistical analyses to determine significance.

**Results:**

Cathepsin B and cathepsin D were detected in IDHWGB by DAB IHC staining. IF IHC staining demonstrated the expression of both cathepsin B and cathepsin D by the OCT4^+^ and SALL4^+^ CSC subpopulations. NanoString gene analysis and CISH confirmed the abundant transcript expression of these cathepsins. The transcriptional and translational expressions of cathepsin G were minimal and were confined to cells within the microvasculature.

**Conclusion:**

This study demonstrated the expression of cathepsin B and cathepsin D but not cathepsin G within the CSC subpopulations of IDHWGB at both the transcriptional and translational level. Cathepsin G was expressed at low levels and was not localized to the CSC population of IDHWGB. The novel finding of cathepsin B and cathepsin D in IDHWGB suggests the presence of bypass loops for the renin-angiotensin system, which may facilitate the production of angiotensin peptides. Elucidating the precise role of these cathepsins may lead to better understanding and more effective treatment of this aggressive tumor.

## Introduction

Glioblastoma multiforme (GBM) is an aggressive primary brain cancer, associated with a 5-year survival rate of 2% (1, 2). Despite intensive treatment comprising surgical resection with postoperative radiotherapy and chemotherapy (3), the median survival remains at 12–15 months (4, 5). The incidence of GBM is bimodal, peaking in the 6th decade, with a lower peak in the 4th decade of life (3, 6, 7).

Recent WHO guidelines for classification of GBM attempt to capture the complex molecular and histological nature of GBM, by separating tumors into two otherwise morphologically indistinguishable categories (7, 8), based on the dysfunction of enzymes involved in the tricarboxylic acid (TCA) cycle, critical for cellular energy production in proliferating tumor cells (7). In GBM, mutations of isocitrate dehydrogenase (IDH), an enzyme catalyzing the oxidative decarboxylation of 2R,3S-isocitrate to 2-oxoglutarate within the TCA cycle, have been observed and are likely to confer an advantage for sustaining tumorigenesis (7, 9). The IDH-wildtype glioblastoma (IDHWGB), mostly prevalent in patients over 55 years, accounts for 90% of cases, while the IDH-mutant type glioblastoma that predominantly occurs in younger patients evolves from a low-grade astrocytoma and is associated with improved survival (7–9).

Cancer stem cells (CSCs), a small population of cells within cancer that possess phenotypic characteristics of embryonic stem cells (ESCs), acquired either from the accumulation of mutations in normal ESCs or progenitor cells, are attributed to the poor prognosis associated with GBM (10–12). CSCs were first identified in brain tumors by Singh et al. (13) and have been detected in many cancers including myelogenous leukemia (14), pancreatic cancer (15), breast cancer (16), oral tongue (17), buccal mucosal (18), and lip (19) squamous cell carcinoma.

Our characterization of CSCs in GBM (2) has led us to postulate that CSCs exist within a putative hierarchy, reminiscent of the hierarchy exhibited by normal stem cells (2, 20, 21). We observed relatively low expression of OCT4, a transcription factor critical for the maintenance of ESC pluripotency and self-renewal (22) and a contrastingly ubiquitous expression of SOX2 and SALL4, both vital transcription factors for sustaining the CSCs (22) within GBM (2). We infer the presence of multiple subpopulations of CSCs at differing degrees of differentiation, within GBM. We propose that the relatively less abundant OCT4^+^ subpopulation represents the most primitive CSCs within GBM, which subsequently differentiate to form SOX2^+^/SALL4^+^ progenitor cells (2). Thus, potential treatment for GBM could focus on targeting the most primitive OCT4^+^ CSC subpopulation.

Furthermore, the putative CSC subpopulations within GBM express components of the renin-angiotensin system (RAS) (23). The classical RAS cascade involves the conversion of the precursor angiotensinogen (AGN) into angiotensin I (ATI), by renin. ATI is further cleaved by angiotensin converting enzyme (ACE) to form angiotensin II (ATII), which interacts with ATII receptor 1 (ATIIR1) and ATII receptor 2 (ATIIR2) (23, 24). There is increasing evidence that the dysregulation of the RAS may play a pivotal role in the progression of cancer, including GBM, through actions mediated by the interaction between ATII and ATIIR1 such as induction of angiogenesis, promotion of cellular proliferation, and inhibition of apoptosis (25, 26). This suggests that CSCs may be a novel therapeutic target by modulation of the RAS (23).

**Figure 1:**
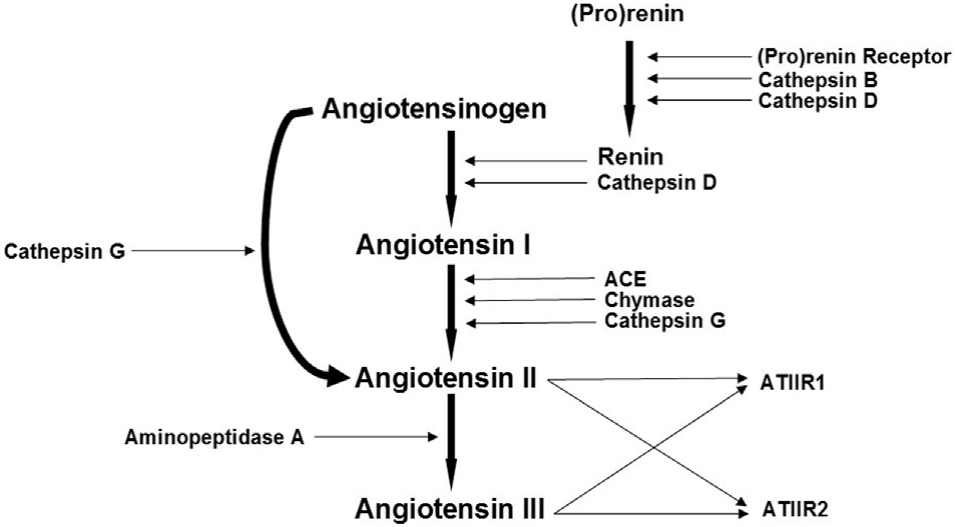
**A schema demonstrating the proposed role of cathepsins B, D, and G, as bypass loops for the renin-angiotensin system (RAS)**. In the RAS, (pro)renin activation is caused by its binding to the (pro)renin receptor. Renin coverts angiotensinogen (AGN) to angiotensin I (ATI) that is acted upon by angiotensin converting enzyme (ACE) to produce angiotensin II (ATII). Aminopeptidase A converts ATII to angiotensin III (ATIII). ATII and ATIII act on angiotensin II receptor 1 (ATIIR1) and angiotensin II receptor 2 (ATIIR2). Cathepsins B, D, and G and chymase are also renin-activating enzymes. Cathepsins B and D also catalyze the conversion of pro(renin) into active renin; cathepsin D converts AGN to ATI; chymase converts ATI to ATII; while cathepsin G promotes generation of ATII from ATI and directly from AGN. Adapted with permission from *Integrative Cancer Science and Therapeutics* (32).

The RAS is highly complex involving many other signaling pathways and enzymes such as cathepsins B, D, and G (27–32) (Figure 1). We have recently identified the presence of cathepsin B, a lysosomal cysteine protease that catalyses the conversion of pro-renin into active rennin (27, 28), cathepsin D, an aspartic lysosomal protease bearing significant homology to renin thus engages in renin-like actions of converting AGN to ATI (29, 30), and cathepsin G, a serine protease with the capacity to generate ATII from ATI and directly from AGN (31, 33), within proliferating infantile hemangioma (IH) (34). These cathepsin isozymes catalyze the production of angiotensin peptides without involvement of classical RAS cascade mechanisms and offer a potential explanation for the variable response of IH to treatment using RAS modulators such as β-blockers and ACE inhibitors (31).

The expression of cathepsin B has previously been identified in GBM tumor cells (35), specifically the population of glioma initiating CSCs (36). Although the role of cathepsin B in GBM has yet to be elucidated, its role in mediating tumor invasion through its proteolytic actions on the extracellular matrix has been proposed (35), while more recent findings suggest that cathepsin B may be critical in imbuing these glioma initiating cells with a stem cell-like phenotype, with characteristics including aberrant self-renewal and proliferation, through upregulation of SOX2 and Bmi1 (36). Cathepsin D has also been observed in numerous cancers, including gliomas, with significantly elevated levels of expression in more aggressive gliomas, such as GBM, implying that serum cathepsin D levels could be a prognostic marker for GBM (37). As studies have observed a positive correlation between levels of cathepsin B and cathepsin D and cancer prognosis, it is likely that both these cathepsins play a fundamental role in contributing to the invasive infiltrative nature and high rates of recurrence in GBM, possibly through their proteolytic actions on components of the extracellular matrix (35, 37).

We hypothesized that cathepsins B, D, and G are expressed by the CSC subpopulations within IDH-negative GBM. In this study, we investigated the presence of these isozymes at the translational level using immunohistochemical (IHC) staining and at the transcriptional level using NanoString gene analysis and colorimetric *in situ* hybridization (CISH).

## Materials and Methods

### Tissue Samples

Six IDH-wildtype glioblastoma (IDHWGB) tissue samples from three male and three female patients aged 42–81 (mean, 64.2) years were sourced from the Gillies McIndoe Research Institute Tissue Bank for this study, which was approved by the Central Regional Health and Disability Ethics Committee (ref. no 15CEN28). Written informed consent was obtained from patients included in this study.

### Histochemical and IHC Staining

Hematoxylin and eosin (H&E) staining was performed on 4μm-thick formalin-fixed paraffin-embedded sections of GBM samples from six patients, to confirm the diagnosis of IDHWGB by an anatomical pathologist (HDB). 3,3-Diaminobenzidine (DAB) and immunofluorescent (IF) IHC staining were then performed, as previously described (2, 23), on the same GBM samples using the Leica Bond Rx auto-stainer (Leica, Nussloch, Germany) with the primary antibodies: cathepsin B (1:1000; cat# sc-6490-R, Santa Cruz, CA, USA), cathepsin D (1:200; cat# NCL-CDm, Leica), and cathepsin G (1:200; cat# sc-33206, Santa Cruz). All antibodies were diluted with Bond™ primary antibody diluent (cat# AR9352, Leica).

To further characterize the expression of cathepsins B, D, and G, two representative samples of IDHWGB from the original cohort of six patients included in DAB IHC staining were selected for IF IHC staining. Dual IF IHC staining was performed using identical primary antibodies and concentrations as for DAB IHC staining, in conjunction with primary antibodies for tryptase (ready-to-use; cat# PA0019, Leica), OCT4 (1:200; cat# NBPI-47923, Novus Biologicus, Littleton, CO, USA) and SALL4 (1:30; cat#6E3, Cell Marque, Rocklin, CA, USA). An appropriate fluorescent secondary antibody of Vectafluor Excel anti-mouse (ready-to-use; cat# VEDK2488, Vector Laboratories, Burlingame, CA, USA) or Alexa Fluor anti-rabbit 594 (1:500; cat# A21207, Life Technologies, Carlsbad, CA, USA) was used for detection.

DAB IHC-stained slides were mounted in Surgipath Micromount mounting medium (cat# 3801732, Leica). IF IHC-stained slides were mounted in Vectashield HardSet anti-fade mounting medium and counter-stained with 4′6-diamino-2-phenylinodole (cat# H-1500, Vector Laboratories).

Positive controls were demonstrated on human placenta for cathepsin B, human breast tissue for cathepsin D, and mouse bone marrow for cathepsin G. Negative controls for DAB IHC staining were performed on sections of IDHWGB using a matched isotype control for both mouse (ready-to-use; cat# IR750, Dako, Copenhagen, Denmark) and rabbit (ready-to-use; cat# IR600, Dako) primary antibodies, to determine the specificity of the amplification cascade. Negative controls for IF IHC staining were performed using a section of GBM tissue with the combined use of primary isotype mouse (ready-to-use; cat# IR750, Dako) and rabbit (ready-to-use; cat# IR600, Dako) antibodies.

### Image Analysis

DAB IHC-stained slides were viewed and the images were captured using the Olympus BX53 light microscope fitted with an Olympus DP21 digital camera and processed with the CellSens 2.0 Software (Olympus, Tokyo, Japan). IF IHC-stained slides were visualized and imaged using the Olympus FV1200 biological confocal laser-scanning microscope and processed with CellSens Dimension 1.11 software using 2D deconvolution algorithm (Olympus).

### Nanostring Gene Expression Analysis

RNA extracted separately from ~20 mg of snap-frozen IDHWGB tissues obtained from six patients was used for NanoString nCounter™ Gene Expression Assay (Nanostring Technologies, Seattle, WA, USA), as previously described (2, 23). Probes for the genes encoding cathepsin B (NM_001908.2), cathepsin D (NM_001909.3), and cathepsin G (NM_001911.2) and the housekeeping gene PGK1 (NM_000291.3) were designed and synthesized by NanoString Technologies. Raw data were analyzed using nSolver™ software (NanoString Technologies) using standard settings and normalized against the housekeeping gene.

### Colorimetric *In Situ* Hybridization

About 4 μm-thick formalin-fixed paraffin-embedded sections of IDHWGB tissues (*n* = 3) selected from the original cohort of six patients were used for mRNA CISH staining, using the Leica Bond Rx autostainer and detected using the ViewRNA red stain kit (Affymetrix, Santa Clara, CA, USA). Probes for cathepsin B (NM_001908), cathepsin D (NM_001909), and cathepsin G (NM_001911) were obtained from Affymetrix. Human placenta, human breast tissue, and mouse bone marrow were used as positive controls for cathepsin B, cathepsin D, and cathepsin G, respectively. Negative controls were exhibited on sections of GBM tissue using a probe for *Bacillus* (cat# VF1-11712, Affymetrix).

### Cell Counting and Statistical Analyses

Using the Olympus BX53 light microscope fitted with an Olympus DP21 digital camera, six fields of view were selected at 400× magnification from each of the six IDHWGB samples included in DAB IHC staining. Fields of view were selected from regions of tumor exhibiting the highest density of staining. Results obtained from cell counting of DAB IHC-stained slides were subjected to the χ^2^ test, to compare the significance of the level of expression for each cathepsin relative to other cathepsins. Data obtained from NanoString gene analysis were subjected to *t*-test using the software SPSS version 22.

## Results

### Histochemical and DAB IHC Staining

**Figure 2:**
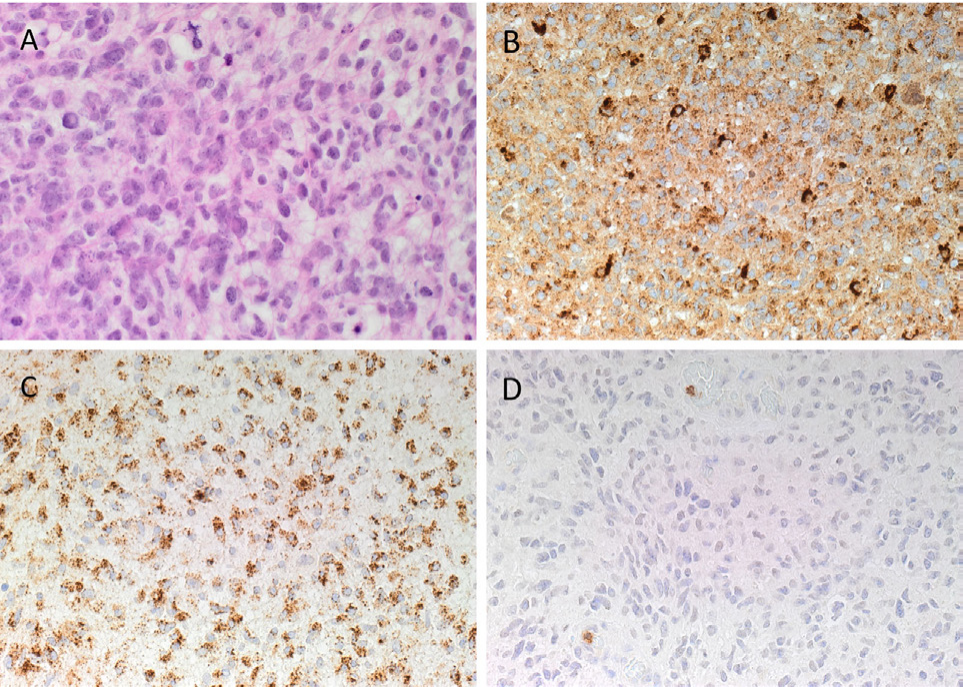
**Representative hematoxylin and eosin stained slide of isocitrate dehydogenase-wildtype glioblastoma sample (A)**. 3,3-Diaminobezidine immunohistochemical-stained slides of representative glioblastoma multiforme sample showing ubiquitous cytoplasmic staining of cathepsin B [**(B)**, brown] and cathepsin D [**(C)**, brown]. Cathepsin G [**(D)**, brown] was localized to cells within the microvessels but not the tumor cells. Nuclei were counter-stained with hematoxylin [**(A–D)**, blue]. Original magnification: 400×.

Hematoxylin and eosin staining (Figure 2A) confirmed the diagnosis of IDHWGB for all six tissue samples. All DAB IHC-stained sections exhibited ubiquitous, granular, cytoplasmic staining for cathepsin B (Figure 2B, brown) and cathepsin D (Figure 2C, brown) at varying intensities. Cytoplasmic staining of cathepsin G (Figure 2D, brown) presents in few cells located within the microvessels within the immediate vicinity of the tumor that did not express the protein.

Positive staining was demonstrated on human placenta tissue for cathepsin B (Figure S1A in Supplementary Material, brown), human breast tissue for cathepsin D (Figure S1B in Supplementary Material, brown), and mouse bone marrow for cathepsin G (Figure S1C in Supplementary Material, brown). Minimal staining was present on the negative control, which was performed on a section of IDHWGB tissue using a matched isotype control for both mouse and rabbit primary antibodies (Figure S1D in Supplementary Material, brown).

### IF IHC Staining

**Figure 3:**
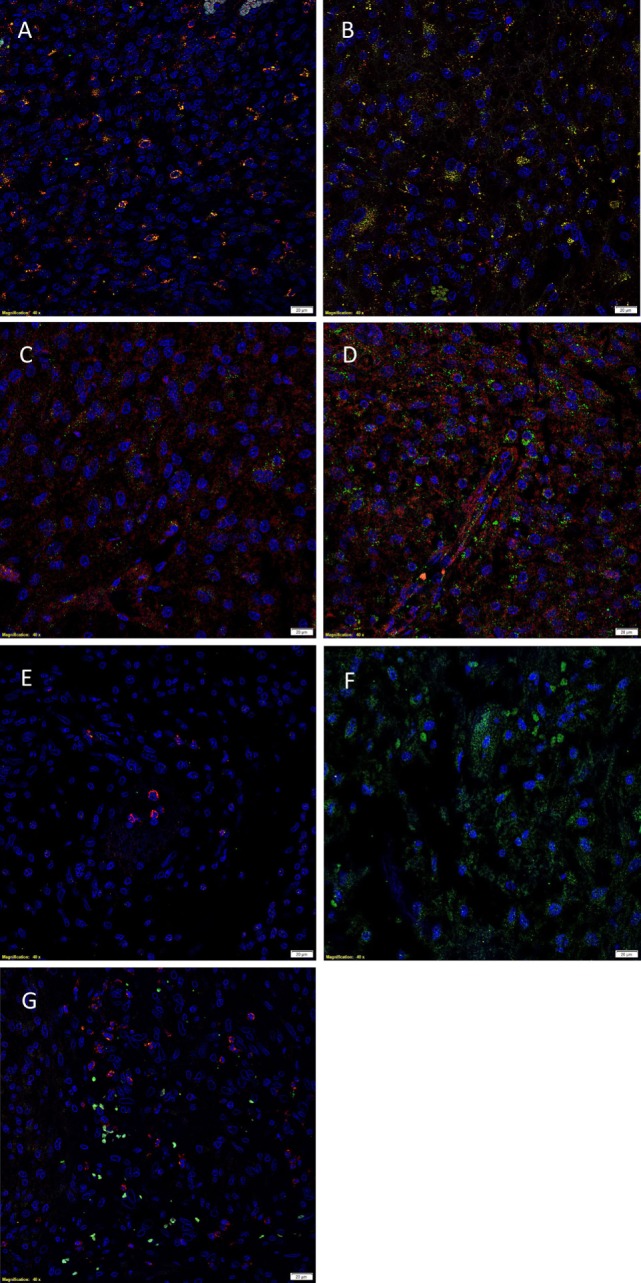
**Representative immunofluorescent immunohistochemical-stained sections of isocitrate dehydogenase-wildtype glioblastoma sample demonstrating the coexpression of cathepsin B [(A,B), red] with the OCT4^+^ [(A), green] and SALL4^+^ [(B), green] cancer stem cell (CSC) subpopulations**. Cathepsin D [**(C,D)**, red] was similarly expressed on the OCT4^+^ [**(C)**, green] and SALL4^+^ [**(D)**, green] CSC subpopulation. Cathepsin G [**(E,F)**, red] was not expressed by the OCT4^+^ [**(E)**, green] and SALL4^+^ [**(F)**, green] CSC subpopulations. Cathepsin G [**(G)**, red] was not expressed by the tryptase^+^ [**(G)**, green] cells. Cell nuclei [**(A–G)**, blue] are displayed by 4′6′-diamidino-2-phenylindole staining. Scale bars: 20 µm.

Immunofluorescent IHC staining showed that cathepsin B (Figures 3A,B, red) was expressed on the OCT4^+^ (Figure 3A, green) and SALL4^+^ (Figure 3B, green) CSC subpopulation. Cathepsin D (Figures 3C,D, red) was also localized to the OCT4^+^ (Figure 3C, green) and SALL4^+^ (Figure 3D, green) CSC subpopulation. However, expression of cathepsin G (Figures 3E,F, red) was localized to cells within the microvessels but was not expressed by the OCT4^+^ (Figure 3E, green) and the SALL4^+^ (Figure 3F, green) CSC subpopulations. Interestingly, cathepsin B (Figure 3A, red) and cathepsin D (Figure 3C, red) were also localized to a separate subpopulation of cells that did not express OCT4 (Figures 3A,C, green). Dual staining of cathepsin G (Figure 3G, red) with tryptase (Figure 3G, green), a common marker of mast cells (34), showed no coexpression of the two proteins.

Images illustrating the individual stains demonstrated in Figure 3 are presented in Figure S2 in Supplementary Material. Minimal staining was present on the negative control (Figure S2O in Supplementary Material), confirming the specificity of the primary antibodies used.

### Nanostring Gene Expression Analysis

**Figure 4:**
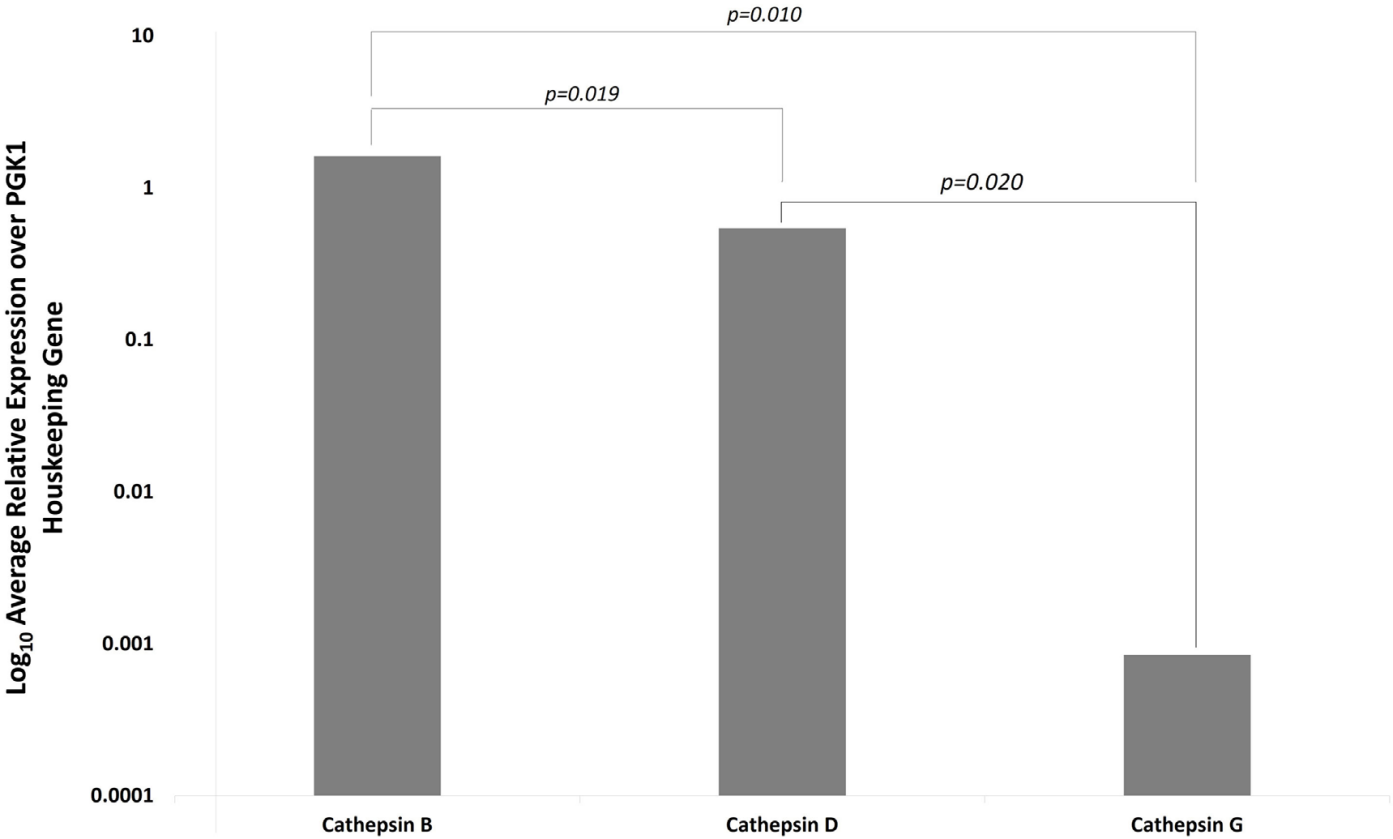
**Expression of cathepsins B, D, and G messenger RNA transcripts in isocitrate dehydogenase-wildtype glioblastoma samples from six patients**. Their expression was normalized over PGK1 housekeeping gene and presented as relative units.

NanoString gene expression analysis was used to investigate the presence of mRNA transcripts for cathepsins B, D, and G with all expression values normalized against the housekeeping gene PGK1. Presence of mRNA coding for cathepsin B and cathepsin D was detected in all six IDHWGB samples, while the corresponding expression of cathepsin G was present in only four of the six samples (Figure 4). Statistical analysis of the NanoString data using *t*-test further confirmed that the mean level of expression of cathepsin B was significantly greater than that of cathepsin D (*p* = 0.019) and cathepsin G (*p* = 0.010); and level of expression of cathepsin D was more abundant than that of cathepsin G (*p* = 0.002).

### Colorimetric *In Situ* Hybridization

**Figure 5:**
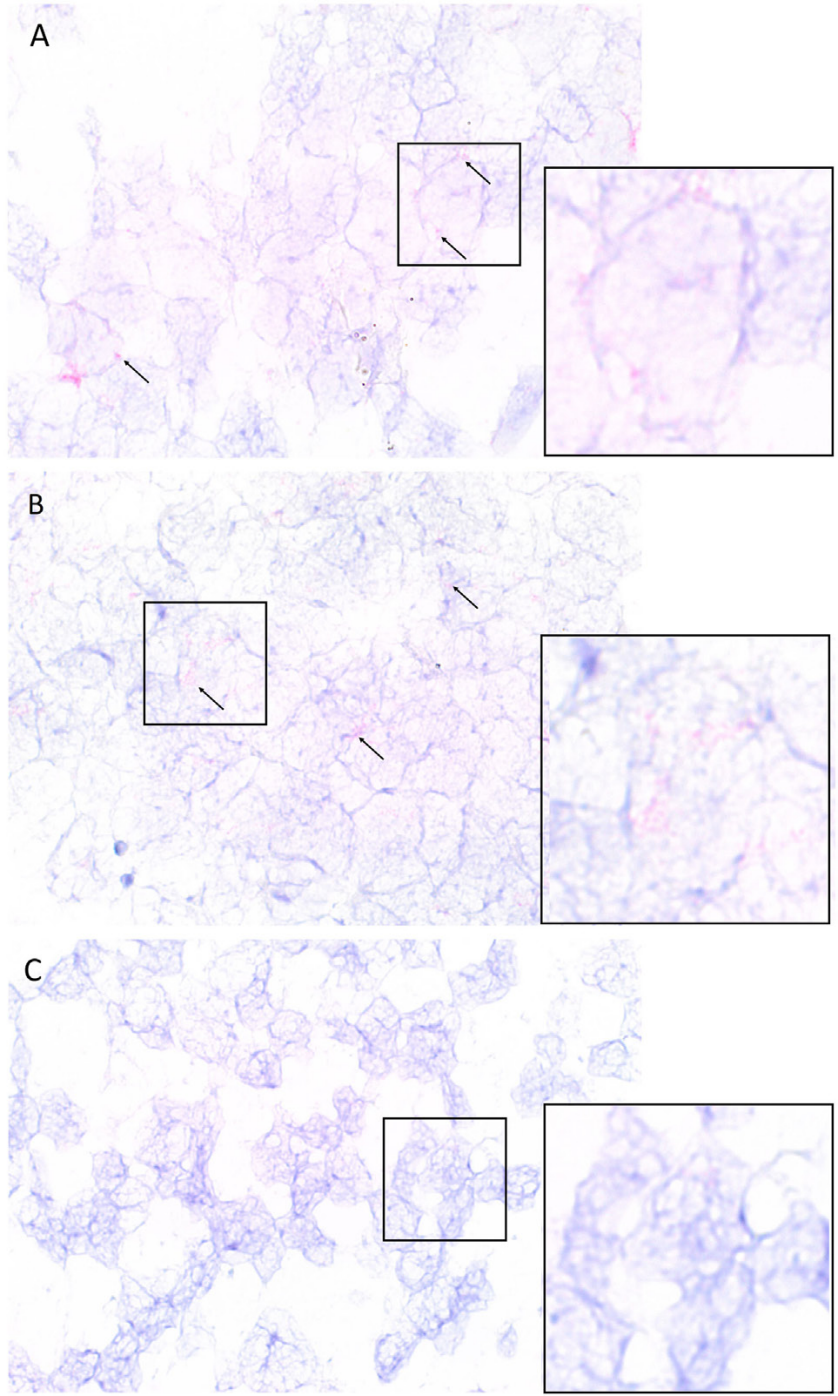
**Representative isocitrate dehydogenase-wildtype glioblastoma samples subjected to colorimetric *in situ* hybridization showing an abundance of cells expressing messenger RNA transcripts of cathepsin B [(A), red] and cathepsin D [(B), red] and minimal expression of cathepsin G [(C), red]**. Nuclei were counter-stained with hematoxylin [**(A–C)**, blue]. Original magnification: 1000×.

Colorimetric *in situ* hybridization demonstrated the presence of mRNA transcripts coding for cathepsin B and cathepsin D within the tumor cells (Figures 5A,B, pink, arrows) in the IDHWGB samples. Minimal transcriptional activation of cathepsin G (Figure 5C, pink, arrows) was observed.

### Cell Counting and Statistical Analyses

**Figure 6:**
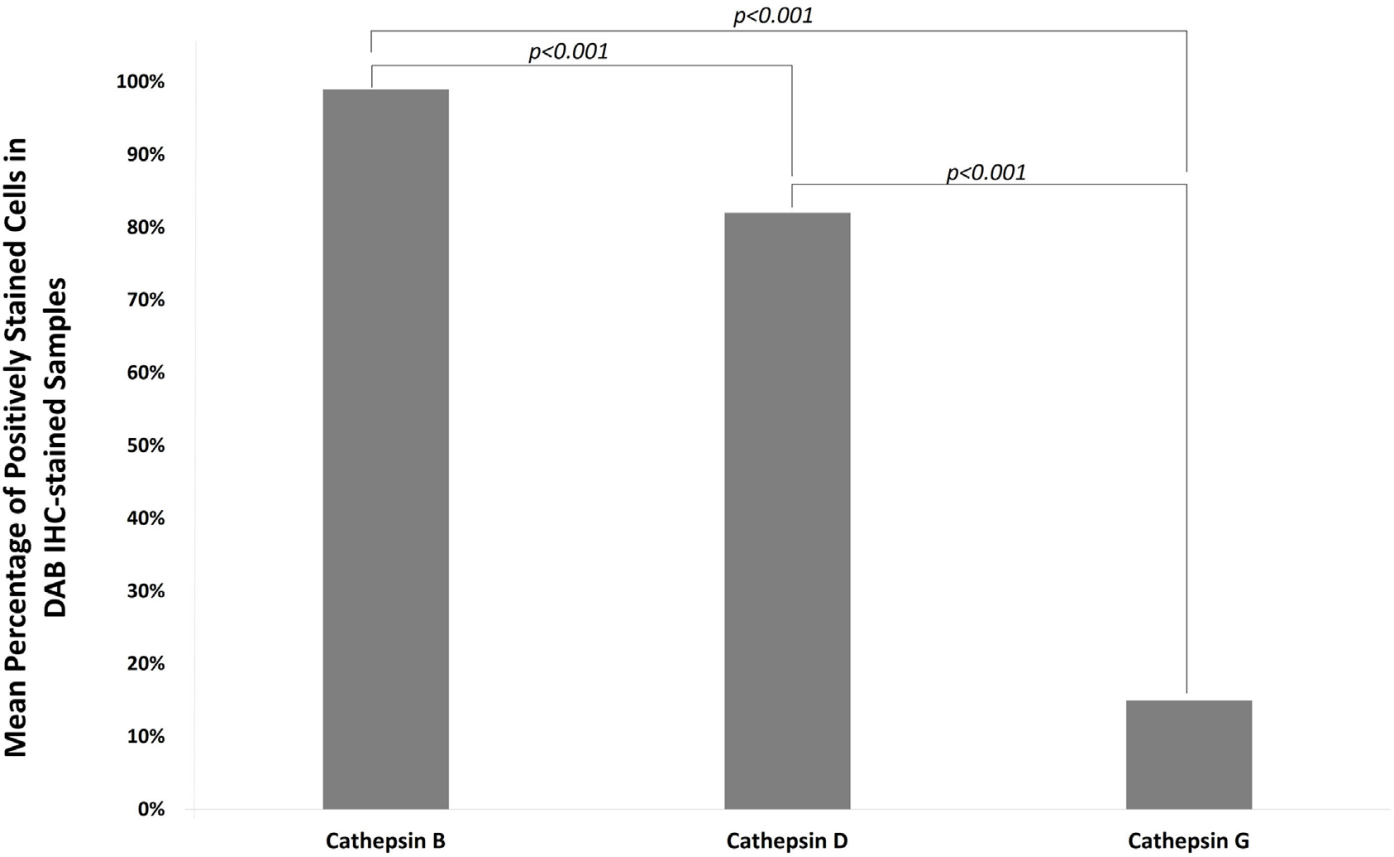
**Mean percentages of expression of cathepsins B, D, and G from cell counting of DAB IHC-stained slides of all IDHWGB samples**. The level of expression of cathepsin B is significantly higher than that of cathepsin D and cathepsin G. The level of expression of cathepsin D is significantly higher than that of cathepsin G.

Data obtained from cell counting of DAB IHC-stained slides of IDHWGB for each cathepsin were analyzed using the chi-square test. The level of expression of cathepsin B (99%) was significantly greater than the level for cathepsin D (82%, *p* < 0.010), while both were much greater than cathepsin G (15%, *p* < 0.001) (Figure 6).

## Discussion

The function of the RAS extends beyond its role in regulating cardiovascular homeostasis. The existence of a paracrine RAS within the local tissue that functions independently or synergistically with the endocrine RAS to maintain systemic and cellular homeostasis has been proposed (33). Dysregulation of the local RAS presumably predisposes cells to malignant transformation through neoplasia-promoting processes mediated through the interaction of ATII with ATIIR1, such as induction of angiogenesis and promotion of cellular proliferation and differentiation (24, 38). Retrospective studies on the relative risk of cancer in patients with hypertension treated with RAS modulators have shown conflicting findings. Lever et al. (38) report reduced incidence of cancer in patients on ACE inhibitors, while other studies have found no significant reduction in risk of breast (39, 40) and prostate (41) cancer. These findings could possibly be explained by the presence of alternative angiotensin peptide generating pathways, such as cathepsins B, D, and G, within the CSCs (33), which imbue them with the ability to evade the modulating effect of the classical RAS.

We have recently demonstrated that CSCs within GBM exhibit a putative hierarchy, reminiscent of normal stem cells, with the OCT4^+^ CSC subpopulation being the most primitive, which we hypothesize proliferate and differentiate to give rise to the SOX2^+^/SALL4^+^ progenitor cells (2). We have further identified the expression of components of the RAS: ATIIR1, PRR, and ACE, by these CSC subpopulations within GBM (23). The new WHO classification system for GBM categorizes these tumors into IDH-wildtype and IDH mutant type glioblastoma (8). The results presented in this study and our recent publication on the characterization of CSCs (2) and their expression of components of the RAS (23) included the same cohort of patients with IDHWGB samples.

The level of expression of cathepsin B and cathepsin D in GBM has been shown to have a positive correlation with tumor aggressiveness and cancer prognosis (35–37), inferring a crucial role for the proteins in the biology of IDHWGB. However, in light of the increasing evidence for the role of CSCs in tumor biology, including IDHWGB, an investigation into the expression of these cathepsins by CSCs in IDHWGB remains to be elucidated. This study demonstrates the abundant expression of cathepsins B and D at both the transcriptional and translational levels and localizing them to the OCT4^+^ and SALL4^+^ CSC subpopulations. The staining patterns for cathepsin B and cathepsin D with intense, granular cytoplasmic staining with regional variations in the intensity were consistent with previous studies (35, 37). Considering the putative hierarchical CSC concept of IDHWGB (2, 20), it is exciting to speculate that both cathepsin B and cathepsin D are expressed by the most primitive OCT4^+^ CSC and maintains throughout its maturation.

Overexpression of cathepsin B has been observed in numerous types of cancers (42, 43), including in gliomas (36, 37, 44). It has been suggested that the proteolytic action of cathepsin B contributes to the infiltrative nature of GBM by destroying components of the extracellular matrix, enabling detachment and infiltration of tumor cells into surrounding tissues (42, 43). Our finding of the localization of cathepsin B to the CSC subpopulations of IDHWGB is consistent with a recent report by Gopinath et al. (36). Upregulation of cathepsin B and uPAR in CSCs are believed to be critical in imbuing these primitive cells with ESC-like characteristics such as the capacity for self-renewal and aberrant growth, through the activation of the sonic hedgehog (SHH) signaling cascade (36). This ultimately results in increased expression of SOX2, a transcription factor responsible for bequeathing stem cells with the capacity for unrestricted self-renewal and proliferation (36) and upregulated in progenitor CSCs in GBM (2) and Bmi1, a protein implicated in brain development and contributes to the aberrant self-renewal and proliferative nature of glioma stem cells (36). Furthermore, inhibition of uPAR and cathepsin B in glioma stem cells was associated with decreased expression of SOX2 and Bmi1, suggesting that cathepsin B and uPAR have pivotal roles in maintaining the malignant nature of CSCs in gliomas (36).

Upregulation of cathepsin D has also been observed within GBM tumor cells (37). While the role of cathepsin D in GBM remains unclear, it has similar proteolytic actions on the extracellular matrix as cathepsin B, and a positive correlation between the expression level of cathepsin D and glioma grade has been observed (37). Furthermore, the use of antibodies targeting cathepsin D has been shown to inhibit GBM invasion in a dose-dependent manner (45), suggesting a critical role for cathepsin D in GBM.

Interestingly, cathepsin G was expressed at low levels in the IDHWGB samples examined at both the gene and protein levels and localizing to cells within the microvessels. Although mast cells commonly contain cathepsin G within their granules (34, 46), our IF IHC staining showed that the cathepsin G^+^ cells did not express tryptase. The lack of detection of tryptase could be explained by the presence of multiple subsets of mast cells, each expressing varying levels and combinations of chymase, tryptase, and cathepsin G within their granules (46, 47). Furthermore, cathepsin G is also expressed by an independent population of circulating cells within IDHWGB.

While the precise roles of cathepsin B and cathepsin D in carcinogenesis, and particularly in the biology of IDHWGB, remain to be conclusively determined, we speculate that they may function as the RAS bypass loops, contributing to the generation of RAS peptides such as ATII, to promote proliferation and differentiation of CSCs in IDHWGB, although further work is required to confirm this.

### Limitations

1. Data from this descriptive study including a relatively small cohort of samples provide a useful foundation for larger future studies.
2. Functional data on the role of cathepsins B, D, and G in IDHWGB and other forms of glioblastoma is the topic of further investigation.
3. Future larger studies on IDHWGB and other forms of glioblastoma may improve the understanding of this heterogenous group of tumors.

## Ethics Statement

This study was approved by the Central Health and Disabilities Ethics Committee (ref. no. 15CEN28).

## Author Contributions

TI and ST formulated the study hypothesis. TI, AW, and ST designed the study. SK, AW, HB, ST, and TI interpreted the DAB and IF IHC and the CISH data. SK, AW, ST, and TI interpreted the NanoString gene expression analysis data. SK performed cell counting on DAB IHC-stained slides. RM conducted statistical analysis and interpreted the results. SK, AW, ST, and TI drafted the manuscript. All authors commented on and approved the manuscript.

## Conflict of Interest Statement

TI and ST are inventors of the PCT patent application (No. PCT/NZ2015/050108) Cancer Diagnosis and Therapy and Cancer Therapeutic (62/452479). The other authors declare that the research was conducted in the absence of any commercial or financial relationships that could be construed as a potential conflict of interest.

## Supplementary Material

Figure S1 **Positive controls of DAB IHC staining for cathepsin B [(A), brown], cathepsin D [(B), brown], and cathepsin G [(C), brown] demonstrated on sections of human placenta, human breast tissue, and mouse bone marrow, respectively**. Negative control **(D)** performed on a section of IDHWGB confirmed specificity of the secondary antibody. Nuclei were counter-stained with hematoxylin [**(A–D)**, blue]. Original magnification: 400×.

Figure S2 **Split images of IF IHC staining of IDHWGB sections shown in** Figure 2. Sections co-stained for OCT4 [**(A,E,I)**, green] and cathepsin B [**(B)**, red], cathepsin D [**(F)**, red], and cathepsin G [**(J)**, red]; SALL4 [**(C,G,K)**, green] and cathepsin B [**(D)**, red], cathepsin D [**(H)**, red] or cathepsin G [**(L)**, red]; and tryptase [**(M)**, green] and cathepsin G [**(N)**, red]. A negative control **(O)** to test the specificity of the fluorescent secondary antibodies is performed on a section of GBM. Cell nuclei [**(A–O)**, blue] are displayed by 4′6′-diamidino-2-phenylindole staining Scale bars: 20 µm.

Figure S3 **Positive controls for CISH staining for cathepsin B [(A), pink], cathepsin D [(B), pink], and cathepsin G [(C), pink] demonstrated on sections of human placenta, human breast tissue, and mouse bone marrow, respectively**. Negative control **(D)** performed on a section of IDHWGB confirms specificity of the secondary antibody. Nuclei were counter-stained with hematoxylin [**(A–D)**, blue]. Original magnification: 400×.
