# Supplementary figures and images for: Expression of Cathepsins B, D, and G in Isocitrate Dehydrogenase-Wildtype Glioblastoma
Source: Front Surg. 2017 May 29;4:28. doi: 10.3389/fsurg.2017.00028 (PMC5447023; doi:10.3389/fsurg.2017.00028)

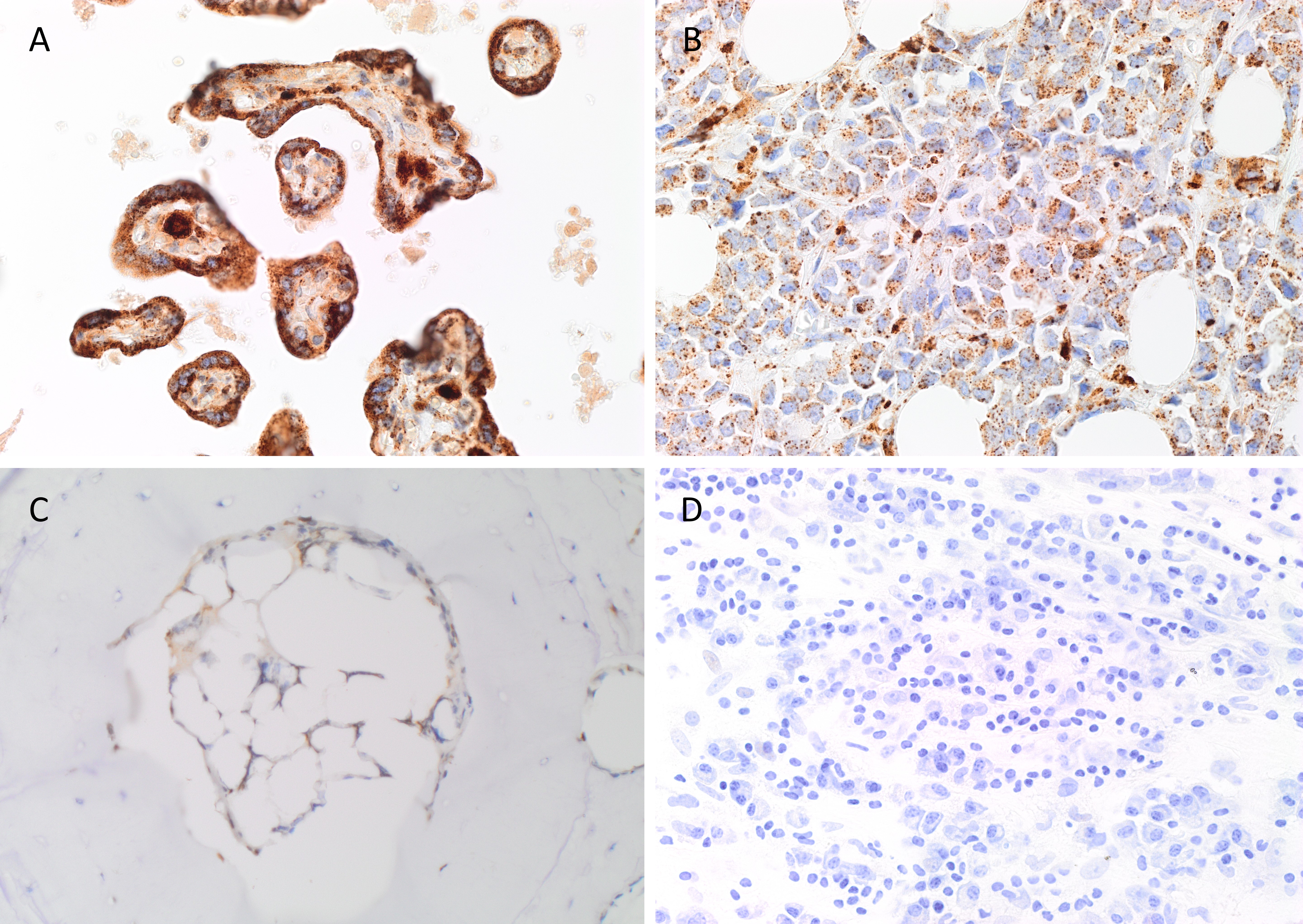

Supplement: Figure S1 — Positive controls of DAB IHC staining for cathepsin B [(A), brown], cathepsin D [(B), brown], and cathepsin G [(C), brown] demonstrated on sections of human placenta, human breast tissue, and mouse bone marrow, respectively. Negative control (D) performed on a section of IDHWGB confirmed specificity of the secondary antibody. Nuclei were counter-stained with hematoxylin [(A–D), blue]. Original magnification: 400×. [file Image_1.JPEG]

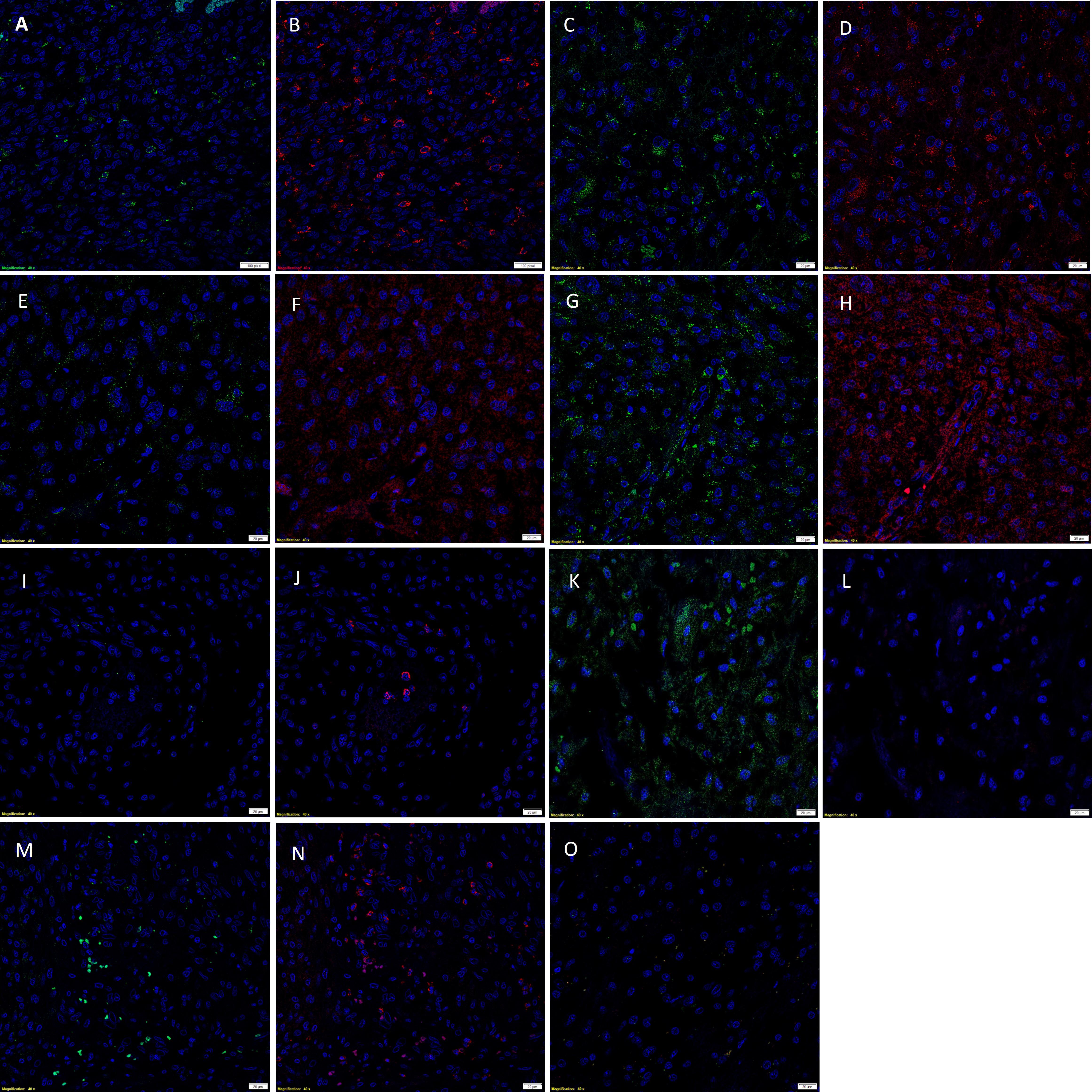

Supplement: Figure S2 — Split images of IF IHC staining of IDHWGB sections shown in Figure 2. Sections co-stained for OCT4 [(A,E,I), green] and cathepsin B [(B), red], cathepsin D [(F), red], and cathepsin G [(J), red]; SALL4 [(C,G,K), green] and cathepsin B [(D), red], cathepsin D [(H), red] or cathepsin G [(L), red]; and tryptase [(M), green] and cathepsin G [(N), red]. A negative control (O) to test the specificity of the fluorescent secondary antibodies is performed on a section of GBM. Cell nuclei [(A–O), blue] are displayed by 4′6′-diamidino-2-phenylindole staining Scale bars: 20 µm. [file Image_2.JPEG]

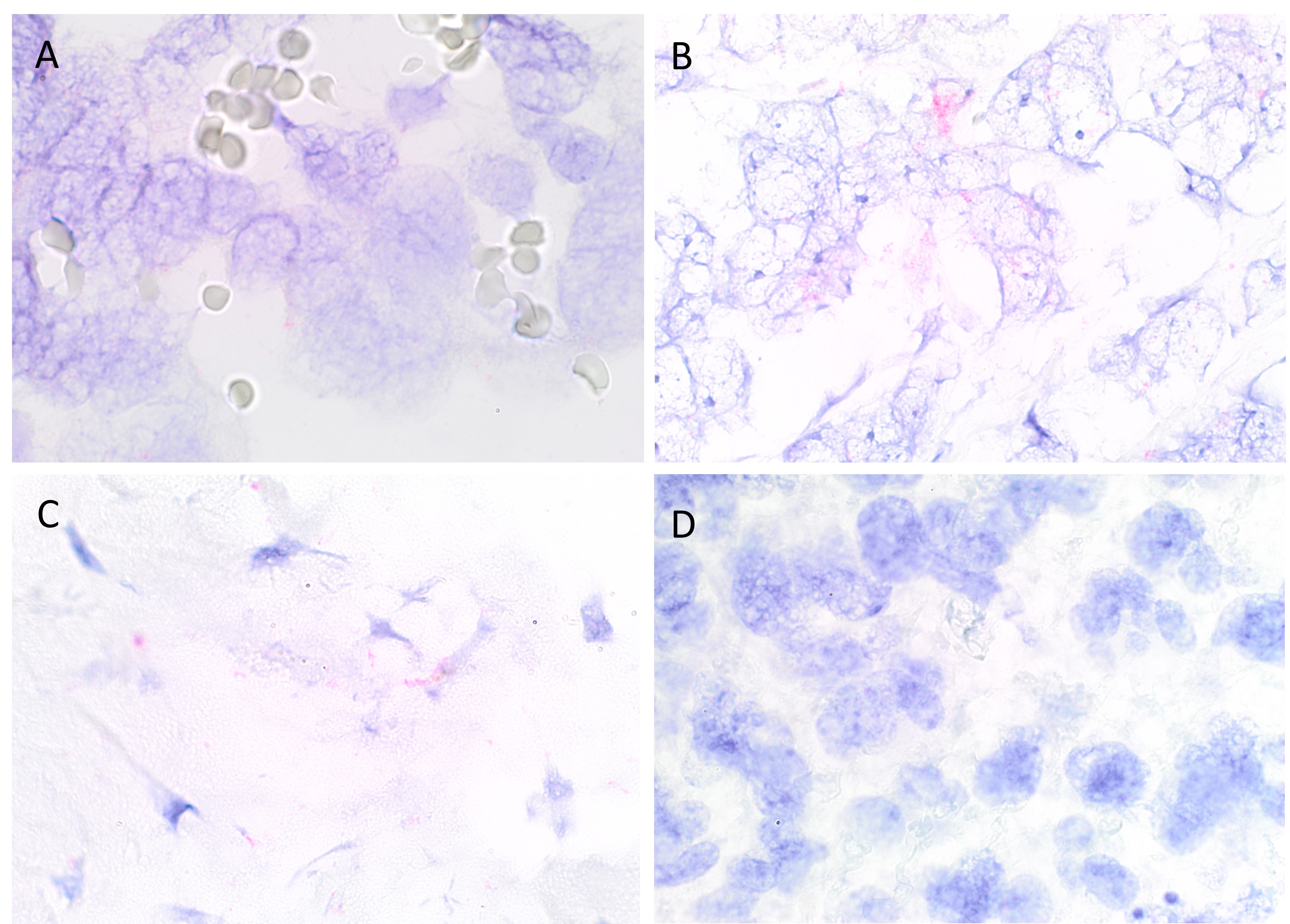

Supplement: Figure S3 — Positive controls for CISH staining for cathepsin B [(A), pink], cathepsin D [(B), pink], and cathepsin G [(C), pink] demonstrated on sections of human placenta, human breast tissue, and mouse bone marrow, respectively. Negative control (D) performed on a section of IDHWGB confirms specificity of the secondary antibody. Nuclei were counter-stained with hematoxylin [(A–D), blue]. Original magnification: 400×. [file Image_3.JPEG]
